# Ethyl 2-acetyl-3-(4-bromo­anilino)butanoate

**DOI:** 10.1107/S1600536809055378

**Published:** 2010-01-09

**Authors:** D. Ravi Kishore Reddy, V.Vijayakumar, J. Suresh, T. Narasimhamurthy, P. L. Nilantha Lakshman

**Affiliations:** a Organic Chemistry Division, School of Advanced Sciences, VIT University, Vellore 632 014, India; b Department of Physics, The Madura College, Madurai 625 011, India; c Materials Research Centre, Indian Institute of Science, Bangalore 560 012, India; d Department of Food Science and Technology, University of Ruhuna, Mapalana, Kamburupitiya 81100, Sri Lanka

## Abstract

The title compound, C_14_H_18_BrNO_3_, adopts an extended conformation, with all of the main-chain torsion angles associated with the ester and amino groups close to *trans*. In the crystal, inversion dimers linked by pairs of N—H⋯O hydrogen bonds are observed.

## Related literature

For related structures see: Rajesh *et al.* (2009 ▶); Priya *et al.* (2006 ▶). For hydrogen-bond motifs, see: Bernstein *et al.* (1995 ▶).
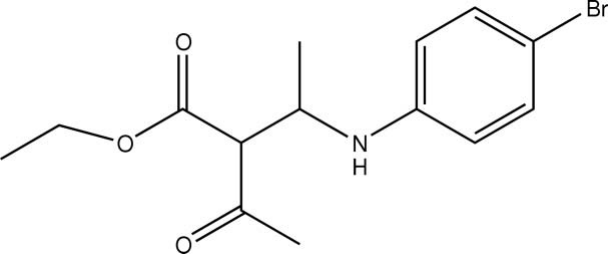


## Experimental

### 

#### Crystal data

C_14_H_18_BrNO_3_
*M* *_r_* = 328.20
Triclinic, 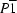

*a* = 6.9107 (3) Å
*b* = 10.1549 (4) Å
*c* = 11.6457 (5) Å
α = 88.104 (2)°
β = 81.932 (2)°
γ = 72.872 (2)°
*V* = 773.26 (6) Å^3^
*Z* = 2
Mo *K*α radiation
μ = 2.66 mm^−1^
*T* = 293 K
0.17 × 0.14 × 0.11 mm

#### Data collection

Bruker SMART APEX CCD diffractometer
Absorption correction: multi-scan (*SADABS*; Bruker, 1998 ▶) *T*_min_ = 0.646, *T*_max_ = 0.746
11063 measured reflections
3140 independent reflections
2298 reflections with *I* > 2σ(*I*)
*R*_int_ = 0.018

#### Refinement

*R*[*F* ^2^ > 2σ(*F* ^2^)] = 0.032
*wR*(*F* ^2^) = 0.084
*S* = 1.06
3140 reflections
179 parameters
H atoms treated by a mixture of independent and constrained refinement
Δρ_max_ = 0.46 e Å^−3^
Δρ_min_ = −0.36 e Å^−3^

### 

Data collection: *SMART* (Bruker, 2001 ▶); cell refinement: *SAINT* (Bruker, 2001 ▶); data reduction: *SAINT*; program(s) used to solve structure: *SHELXS97* (Sheldrick, 2008 ▶); program(s) used to refine structure: *SHELXL97* (Sheldrick, 2008 ▶); molecular graphics: *PLATON* (Spek, 2009 ▶); software used to prepare material for publication: *SHELXL97*.

## Supplementary Material

Crystal structure: contains datablocks global, I. DOI: 10.1107/S1600536809055378/ci5002sup1.cif

Structure factors: contains datablocks I. DOI: 10.1107/S1600536809055378/ci5002Isup2.hkl

Additional supplementary materials:  crystallographic information; 3D view; checkCIF report

## Figures and Tables

**Table 1:** Hydrogen-bond geometry (Å, °)

*D*—H⋯*A*	*D*—H	H⋯*A*	*D*⋯*A*	*D*—H⋯*A*
N7—H7⋯O12^i^	0.82 (3)	2.22 (3)	3.025 (2)	169 (3)

Symmetry code: (i) 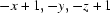.

**Fig. 1.**
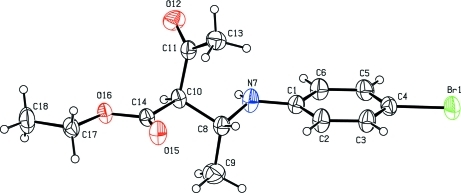
The molecular structure of the title compound, showing 30% probability displacement ellipsoids and the atom-numbering scheme.

**Fig. 2.**
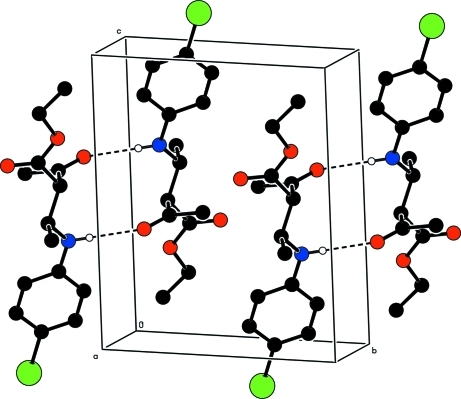
Part of the crystal structure of the title compound, showing hydrogen-bonded dimers. H atoms not involved in hydrogen-bonding (dashed lines) have been omitted for clarity

## supplementary crystallographic information

### Comment

Crystal structures of ethyl 2-acetyl-3-(4-chloroanilino)butanoate (Rajesh *et al.*, 2009) and 2-acetyl-3-anilinobutanoate (Priya *et al.*, 2006) have been reported. Now, we report here the the crystal structure of the title compound.

In the title molecule (Fig. 1), there are three planar subunits *viz*., the bromophenyl amine (C1–C6/Br1/N7), acetyl (C10/C11/O12/C13) and ethyl acetate (C10/C14/O15/O16/C17/C18) groups. The bromophenyl amino ring is inclined at angles of 77.5 (1) and 4.9 (1)° to the acetyl and ethyl acetate groups, respectively, with the acetyl group at an angle of 72.6 (1)° to the ethyl acetate group. The molecules adopts an extended conformation, with all of the main chain torsion angles associated with the ester and amino groups, *i.e.* from C18—C17—O16—C14 to C10—C8—N7—C1 lie in the range -144.14 (19)–179.9 (2)°.

In the crystal structure, molecules associate into dimers through intermolecular N—H···O hydrogen bonds (Table 1). The hydrogen-bonded centrosymmetric dimers are characterized by an *R*_2_^2^(12) ring motif (Fig. 2) (Bernstein *et al.*, 1995).

### Experimental

A mixture of acetaldehyde (22.5 ml), ethyl acetoacetate (6.3 ml) and 4-bromoaniline (8.7 ml) was placed in a round bottomed flask. The contents were stirred at 273 K to 278 K for 5 h under nitrogen atmosphere. A paste-like solid was formed, which was initially washed with benzene, then chloroform and then extracted with diethyl ether. The extract allowed to evaporate under room temperature yielded the product with crystalline nature. The resulting compound was recrystallized from diethyl ether (yield: 86%, m.p. 349 K).

### Refinement

The amino H atom was located in a difference map and was refined isotropically. The remaining H atoms were placed in calculated positions and allowed to ride on their carrier atoms, with C–H = 0.93–0.98 Å and *U*_iso_ = 1.2*U*_eq_(C,) for CH, CH_2_ groups and *U*_iso_ = 1.5*U*_eq_(C) for CH_3_ groups.

### Crystal data

**Table:**

C_14_H_18_BrNO_3_	*Z* = 2
*M**_r_* = 328.20	*F*(000) = 336
Triclinic, *P*1	*D*_x_ = 1.410 Mg m^−^^3^
Hall symbol: -P 1	Mo *K*α radiation, λ = 0.71073 Å
*a* = 6.9107 (3) Å	Cell parameters from 25 reflections
*b* = 10.1549 (4) Å	θ = 2–25°
*c* = 11.6457 (5) Å	µ = 2.66 mm^−^^1^
α = 88.104 (2)°	*T* = 293 K
β = 81.932 (2)°	Block, colourless
γ = 72.872 (2)°	0.17 × 0.14 × 0.11 mm
*V* = 773.26 (6) Å^3^	

### Data collection

**Table:**

Bruker SMART APEX CCD diffractometer	3140 independent reflections
Radiation source: fine-focus sealed tube	2298 reflections with *I* > 2σ(*I*)
graphite	*R*_int_ = 0.018
ω scans	θ_max_ = 26.5°, θ_min_ = 2.1°
Absorption correction: multi-scan (*SADABS*; Bruker, 1998)	*h* = −8→8
*T*_min_ = 0.646, *T*_max_ = 0.746	*k* = −12→12
11063 measured reflections	*l* = −14→13

### Refinement

**Table:**

Refinement on *F*^2^	Primary atom site location: structure-invariant direct methods
Least-squares matrix: full	Secondary atom site location: difference Fourier map
*R*[*F*^2^ > 2σ(*F*^2^)] = 0.032	Hydrogen site location: inferred from neighbouring sites
*wR*(*F*^2^) = 0.084	H atoms treated by a mixture of independent and constrained refinement
*S* = 1.06	*w* = 1/[σ^2^(*F*_o_^2^) + (0.0399*P*)^2^ + 0.2034*P*] where *P* = (*F*_o_^2^ + 2*F*_c_^2^)/3
3140 reflections	(Δ/σ)_max_ = 0.002
179 parameters	Δρ_max_ = 0.46 e Å^−^^3^
0 restraints	Δρ_min_ = −0.36 e Å^−^^3^

### Special details

**Table:**

Geometry. All e.s.d.'s (except the e.s.d. in the dihedral angle between two l.s. planes) are estimated using the full covariance matrix. The cell e.s.d.'s are taken into account individually in the estimation of e.s.d.'s in distances, angles and torsion angles; correlations between e.s.d.'s in cell parameters are only used when they are defined by crystal symmetry. An approximate (isotropic) treatment of cell e.s.d.'s is used for estimating e.s.d.'s involving l.s. planes.
Refinement. Refinement of *F*^2^ against ALL reflections. The weighted *R*-factor *wR* and goodness of fit *S* are based on *F*^2^, conventional *R*-factors *R* are based on *F*, with *F* set to zero for negative *F*^2^. The threshold expression of *F*^2^ > σ(*F*^2^) is used only for calculating *R*-factors(gt) *etc*. and is not relevant to the choice of reflections for refinement. *R*-factors based on *F*^2^ are statistically about twice as large as those based on *F*, and *R*- factors based on ALL data will be even larger.

### Fractional atomic coordinates and isotropic or equivalent isotropic displacement parameters (Å^2^)

**Table:**

	*x*	*y*	*z*	*U*_iso_*/*U*_eq_	
H7	0.351 (4)	0.090 (3)	0.647 (2)	0.057 (8)*	
C1	0.3126 (3)	0.2211 (2)	0.76505 (19)	0.0455 (5)	
C2	0.2092 (4)	0.3540 (2)	0.8052 (2)	0.0594 (7)	
H2	0.1187	0.4130	0.7609	0.071*	
C3	0.2387 (4)	0.4000 (3)	0.9097 (2)	0.0589 (6)	
H3	0.1704	0.4899	0.9346	0.071*	
C4	0.3687 (4)	0.3134 (3)	0.97710 (19)	0.0501 (6)	
C5	0.4724 (4)	0.1817 (3)	0.9396 (2)	0.0567 (6)	
H5	0.5614	0.1232	0.9850	0.068*	
C6	0.4451 (4)	0.1361 (2)	0.8353 (2)	0.0555 (6)	
H6	0.5164	0.0466	0.8107	0.067*	
C8	0.1297 (3)	0.2327 (2)	0.59098 (19)	0.0479 (5)	
H8	0.0953	0.3333	0.5958	0.058*	
C9	−0.0615 (5)	0.1911 (3)	0.6344 (3)	0.0772 (8)	
H9A	−0.0327	0.0932	0.6259	0.116*	
H9B	−0.1686	0.2372	0.5900	0.116*	
H9C	−0.1041	0.2164	0.7147	0.116*	
C10	0.2161 (3)	0.1881 (2)	0.46537 (18)	0.0420 (5)	
H10	0.2436	0.0880	0.4597	0.050*	
C11	0.4164 (3)	0.2228 (2)	0.42887 (19)	0.0459 (5)	
C13	0.4194 (4)	0.3670 (2)	0.4450 (2)	0.0601 (7)	
H13A	0.4127	0.3852	0.5260	0.090*	
H13B	0.3041	0.4296	0.4155	0.090*	
H13C	0.5434	0.3790	0.4038	0.090*	
C14	0.0662 (3)	0.2561 (2)	0.38162 (18)	0.0426 (5)	
C17	−0.0513 (4)	0.2264 (3)	0.2058 (2)	0.0576 (6)	
H17A	−0.0149	0.3049	0.1698	0.069*	
H17B	−0.1939	0.2569	0.2395	0.069*	
C18	−0.0196 (5)	0.1175 (3)	0.1183 (3)	0.0826 (9)	
H18A	0.1222	0.0870	0.0862	0.124*	
H18B	−0.1008	0.1535	0.0576	0.124*	
H18C	−0.0595	0.0413	0.1542	0.124*	
N7	0.2990 (3)	0.1728 (2)	0.65820 (17)	0.0567 (6)	
O12	0.5679 (3)	0.13442 (17)	0.38785 (16)	0.0664 (5)	
O15	−0.0423 (3)	0.37258 (17)	0.39042 (15)	0.0613 (5)	
O16	0.0767 (2)	0.17047 (15)	0.29562 (13)	0.0491 (4)	
Br1	0.40364 (5)	0.37808 (3)	1.12205 (2)	0.07438 (14)	

### Atomic displacement parameters (Å^2^)

**Table:**

	*U*^11^	*U*^22^	*U*^33^	*U*^12^	*U*^13^	*U*^23^
C1	0.0498 (13)	0.0417 (13)	0.0422 (12)	−0.0068 (10)	−0.0120 (10)	0.0033 (10)
C2	0.0734 (17)	0.0460 (15)	0.0512 (14)	0.0027 (13)	−0.0276 (12)	0.0024 (11)
C3	0.0721 (17)	0.0470 (15)	0.0529 (14)	−0.0056 (12)	−0.0164 (12)	−0.0070 (11)
C4	0.0556 (14)	0.0575 (16)	0.0419 (12)	−0.0201 (12)	−0.0150 (10)	0.0025 (11)
C5	0.0592 (15)	0.0580 (16)	0.0521 (14)	−0.0094 (13)	−0.0246 (12)	0.0105 (12)
C6	0.0604 (15)	0.0440 (14)	0.0561 (14)	−0.0004 (11)	−0.0211 (12)	0.0020 (11)
C8	0.0517 (13)	0.0458 (13)	0.0446 (12)	−0.0076 (11)	−0.0162 (10)	0.0031 (10)
C9	0.0732 (19)	0.097 (2)	0.0654 (18)	−0.0332 (17)	−0.0090 (15)	0.0152 (16)
C10	0.0500 (13)	0.0295 (11)	0.0466 (12)	−0.0067 (9)	−0.0175 (10)	−0.0011 (9)
C11	0.0486 (13)	0.0414 (13)	0.0446 (12)	−0.0045 (11)	−0.0140 (10)	−0.0049 (10)
C13	0.0568 (15)	0.0448 (15)	0.0798 (18)	−0.0173 (12)	−0.0046 (13)	−0.0139 (13)
C14	0.0452 (12)	0.0407 (14)	0.0426 (12)	−0.0104 (11)	−0.0128 (10)	−0.0008 (10)
C17	0.0536 (14)	0.0708 (17)	0.0482 (14)	−0.0101 (13)	−0.0238 (11)	−0.0005 (12)
C18	0.090 (2)	0.094 (2)	0.0631 (18)	−0.0131 (18)	−0.0333 (16)	−0.0209 (16)
N7	0.0715 (14)	0.0405 (13)	0.0493 (12)	0.0057 (11)	−0.0261 (10)	−0.0037 (9)
O12	0.0546 (11)	0.0479 (10)	0.0843 (14)	0.0009 (9)	0.0003 (10)	−0.0136 (9)
O15	0.0705 (11)	0.0435 (10)	0.0609 (10)	0.0071 (9)	−0.0289 (9)	−0.0073 (8)
O16	0.0552 (9)	0.0446 (9)	0.0476 (9)	−0.0076 (7)	−0.0211 (7)	−0.0057 (7)
Br1	0.0865 (2)	0.0964 (3)	0.04916 (17)	−0.03385 (18)	−0.02258 (14)	−0.00476 (14)

### Geometric parameters (Å, °)

**Table:**

C1—N7	1.378 (3)	C10—C14	1.523 (3)
C1—C2	1.390 (3)	C10—C11	1.527 (3)
C1—C6	1.397 (3)	C10—H10	0.98
C2—C3	1.380 (3)	C11—O12	1.210 (3)
C2—H2	0.93	C11—C13	1.489 (3)
C3—C4	1.372 (3)	C13—H13A	0.96
C3—H3	0.93	C13—H13B	0.96
C4—C5	1.371 (3)	C13—H13C	0.96
C4—Br1	1.903 (2)	C14—O15	1.199 (3)
C5—C6	1.370 (3)	C14—O16	1.328 (3)
C5—H5	0.93	C17—O16	1.457 (3)
C6—H6	0.93	C17—C18	1.476 (4)
C8—N7	1.469 (3)	C17—H17A	0.97
C8—C9	1.520 (4)	C17—H17B	0.97
C8—C10	1.530 (3)	C18—H18A	0.96
C8—H8	0.98	C18—H18B	0.96
C9—H9A	0.96	C18—H18C	0.96
C9—H9B	0.96	N7—H7	0.82 (3)
C9—H9C	0.96		
			
N7—C1—C2	123.4 (2)	C11—C10—C8	110.94 (18)
N7—C1—C6	119.2 (2)	C14—C10—H10	108.4
C2—C1—C6	117.3 (2)	C11—C10—H10	108.4
C3—C2—C1	121.0 (2)	C8—C10—H10	108.4
C3—C2—H2	119.5	O12—C11—C13	121.4 (2)
C1—C2—H2	119.5	O12—C11—C10	120.3 (2)
C4—C3—C2	120.2 (2)	C13—C11—C10	118.39 (19)
C4—C3—H3	119.9	C11—C13—H13A	109.5
C2—C3—H3	119.9	C11—C13—H13B	109.5
C5—C4—C3	120.0 (2)	H13A—C13—H13B	109.5
C5—C4—Br1	120.62 (17)	C11—C13—H13C	109.5
C3—C4—Br1	119.42 (19)	H13A—C13—H13C	109.5
C6—C5—C4	120.0 (2)	H13B—C13—H13C	109.5
C6—C5—H5	120.0	O15—C14—O16	124.58 (19)
C4—C5—H5	120.0	O15—C14—C10	124.46 (19)
C5—C6—C1	121.5 (2)	O16—C14—C10	110.93 (18)
C5—C6—H6	119.3	O16—C17—C18	108.5 (2)
C1—C6—H6	119.3	O16—C17—H17A	110.0
N7—C8—C9	113.4 (2)	C18—C17—H17A	110.0
N7—C8—C10	105.24 (18)	O16—C17—H17B	110.0
C9—C8—C10	113.0 (2)	C18—C17—H17B	110.0
N7—C8—H8	108.3	H17A—C17—H17B	108.4
C9—C8—H8	108.3	C17—C18—H18A	109.5
C10—C8—H8	108.3	C17—C18—H18B	109.5
C8—C9—H9A	109.5	H18A—C18—H18B	109.5
C8—C9—H9B	109.5	C17—C18—H18C	109.5
H9A—C9—H9B	109.5	H18A—C18—H18C	109.5
C8—C9—H9C	109.5	H18B—C18—H18C	109.5
H9A—C9—H9C	109.5	C1—N7—C8	124.3 (2)
H9B—C9—H9C	109.5	C1—N7—H7	116.0 (18)
C14—C10—C11	108.83 (18)	C8—N7—H7	112.9 (19)
C14—C10—C8	111.84 (18)	C14—O16—C17	116.17 (17)
			
N7—C1—C2—C3	−175.7 (3)	C8—C10—C11—O12	126.8 (2)
C6—C1—C2—C3	0.7 (4)	C14—C10—C11—C13	70.2 (3)
C1—C2—C3—C4	−1.3 (4)	C8—C10—C11—C13	−53.2 (3)
C2—C3—C4—C5	1.2 (4)	C11—C10—C14—O15	−85.1 (3)
C2—C3—C4—Br1	−179.0 (2)	C8—C10—C14—O15	37.8 (3)
C3—C4—C5—C6	−0.5 (4)	C11—C10—C14—O16	92.9 (2)
Br1—C4—C5—C6	179.66 (19)	C8—C10—C14—O16	−144.14 (19)
C4—C5—C6—C1	−0.1 (4)	C2—C1—N7—C8	−21.6 (4)
N7—C1—C6—C5	176.5 (2)	C6—C1—N7—C8	162.1 (2)
C2—C1—C6—C5	0.0 (4)	C9—C8—N7—C1	−78.0 (3)
N7—C8—C10—C14	−173.30 (18)	C10—C8—N7—C1	158.0 (2)
C9—C8—C10—C14	62.5 (3)	O15—C14—O16—C17	1.5 (3)
N7—C8—C10—C11	−51.6 (2)	C10—C14—O16—C17	−176.53 (19)
C9—C8—C10—C11	−175.8 (2)	C18—C17—O16—C14	179.9 (2)
C14—C10—C11—O12	−109.7 (2)		

### Hydrogen-bond geometry (Å, °)

**Table:**

*D*—H···*A*	*D*—H	H···*A*	*D*···*A*	*D*—H···*A*
N7—H7···O12^i^	0.82 (3)	2.22 (3)	3.025 (2)	169 (3)

Symmetry codes: (i) −*x*+1, −*y*, −*z*+1.
